# Caffeine ingestion before exercise improves prolonged intermittent-sprint performance of team-sport athletes in normobaric hypoxia

**DOI:** 10.3389/fnut.2025.1717009

**Published:** 2026-01-14

**Authors:** Wei Li, Zuojun Cao, Jie Chang, Ruiguo Xue, Jue Liu, Li Guo, Yinhang Cao, Olivier Girard

**Affiliations:** 1 School of Sports and Health Management, Changzhou Vocational Institute of Engineering, Changzhou, China; 2 Department of Rehabilitation Medicine, Huashan Hospital, Fudan University, Shanghai, China; 3 School of Athletic Performance, Shanghai University of Sport, Shanghai, China; 4 School of Exercise and Health, Shanghai University of Sport, Shanghai, China; 5 School of Human Sciences (Exercise and Sport Science), The University of Western Australia, Perth, WA, Australia

**Keywords:** ergogenic aids, hypoxia, nutritional supplement, repeated-sprint, simulated altitude, team-sports

## Abstract

**Purpose:**

To investigate whether caffeine ingestion before exercise enhances performance during a prolonged intermittent-sprint test (IST) under hypoxic conditions simulating team-sport activity.

**Methods:**

In a randomized, double-blind, placebo-controlled crossover design, fifteen (five females) team-sport athletes (21 ± 2 years; peak oxygen uptake: 51.9 ± 3.4 ml/kg/min) completed two IST sessions in normobaric hypoxia (inspired oxygen fraction: 16.5 ± 0.2%), separated by 7 days. Sixty minutes before each session, participants ingested either 6 mg/kg caffeine or a placebo. The IST, performed on a cycle ergometer, consisted of two 40-min halves. Each half included twenty 2-min blocks, comprising an 8-s all-out sprint, 100 s of active recovery at 35% peak oxygen uptake, and 12 s of passive rest.

**Results:**

Total work done was significantly greater with caffeine, increasing by 6.2% in the first half (154 ± 26 vs. 145 ± 27 kJ) and a 5.3% increase in the second half (138 ± 20 vs. 132 ± 22 kJ) compared to placebo (all *p* < 0.05). Caffeine also significantly increased both peak and mean power output (all *p* < 0.05). Blood lactate concentrations were higher in both halves with caffeine, while ratings of perceived exertion and breathing difficulty were significantly lower in the second half compared to placebo (all *p* < 0.05).

**Conclusion:**

A moderate dose of caffeine intake before exercise significantly enhances prolonged intermittent-sprint performance in competitive team-sport athletes under moderate normobaric hypoxia.

## Introduction

The globalization of team-sports (e.g., football/soccer, rugby and field hockey) has made it increasingly common for athletes to compete at low to moderate altitude (1,200–2,500 m) (1–3). For example, some matches of the 23*^rd^* FIFA World Cup will be held in Mexico City at an elevation of 2,300 m. Athletes engaged in these sports must repeatedly perform intermittent, maximal or near-maximal efforts (e.g., accelerations, changes in pace, sprints) over extended periods (1–2 h) (4, 5).

Data from official games played at altitude show a 7%–21% reduction in total distance covered and sprint frequency among sea-level residents compared with matches at sea level (6–8). Laboratory studies support these real-world observations, reporting impaired intermittent sprint performance (e.g., work done, power output) under hypoxic conditions (9, 10). Although sprinting represents only 5%–10% of total distance covered and 1%–3% of match time (11, 12), its strategic importance is high, as straight sprints are the most frequent action leading to goals in football (13). Therefore, developing interventions to enhance prolonged intermittent-sprint performance in hypoxia is particularly relevant for sea-level residents.

Caffeine is widely used by athletes as a convenient, fast-acting ergogenic aid (14). Numerous studies demonstrate its benefits for endurance, resistance exercise, and sprint performance under both normoxic and hypoxic conditions (15–17). In a previous study, we examined the dose-response effects of caffeine on repeated sprint performance in team-sport athletes under hypoxic environments [inspired O_2_ fraction: 16.5% (∼2,000 m simulated altitude)] (18). A moderate dose (6 mg/kg) was most effective, enhancing the number of sprints to exhaustion compared with low (3 mg/kg) and high (9 mg/kg) doses (18). However, this protocol did not replicate the intensity and duration of actual team-sport match play. To better assess the ergogenic effects of moderate caffeine intake, protocols should mirror typical game demands (e.g., 2 × 40-min halves of intermittent, high-intensity effort).

Therefore, this study aimed to assess the performance and physiological responses of moderate caffeine intake (6 mg/kg) during a modified prolonged intermittent-sprint test [IST, Bishop and Claudius (19)] designed to mimic the multiple-sprint patterns and metabolic demands of team-sport match play. We hypothesized that, compared to placebo, caffeine ingestion before exercise would increase total work done and power output during the IST in team-sport athletes.

## Materials and methods

### Participants

*A priori* power analysis conducted using G*Power software (version 3.1; Universität Düsseldorf, Düsseldorf, Germany) indicated that 12 participants would be sufficient [alpha: 0.05, power: 0.80, correlation coefficient: 0.5, and effect size: 0.5 for total work done as reported by Schneiker et al. (20)]. To account for potential dropouts, 15 college athletes (five females) from various team-sports (i.e., soccer, basketball and volleyball) volunteered [age: 21 ± 2 years; body mass: 69.5 ± 5.5 kg; height: 173.3 ± 5.8 cm; peak oxygen uptake (V̇O_2peak_): 51.9 ± 3.4 ml/kg/min]. All were non-smokers and had no altitude training in the previous six months. Habitual caffeine intake (50 ± 46 mg/day) was assessed using a validated self-reported questionnaire (21), and participants were categorized as caffeine-naïve or mild caffeine consumers (0–3 mg/kg/day) (22). Participants maintained their regular diet, daily routines, and weekly training regimens throughout the study. They were instructed to abstain from alcohol and caffeine for >12 h, based on the pharmacokinetics of caffeine (mean elimination half-life ∼3–7 h) to minimize residual acute effects (23), and from strenuous exercise for >12 h preceding each trial. This study received approval from Shanghai University of Sport ethics committee (No. 102772023RT204) and was conducted in accordance with the latest Declaration of Helsinki (except database registration). Each participant provided verbal and written informed consent.

### Experiment design

Participants visited the laboratory on three occasions. During visit 1, they completed an incremental cycling test and familiarization (see section “Assessment of peak oxygen uptake and familiarization session”). They were instructed to record and subsequently replicate their food and fluid intake for 24 h before each visit. At least 48 h later, participants performed two experimental trials (visits 2 and 3) in a double-blind, placebo-controlled, randomized design, with sessions separated by 5–7 days (18, 20). An independent researcher used randomization software (Excel Office, Microsoft, Washington, United States) to randomize and counterbalance the order of intervention (17). All trials were held at the same time of day under controlled conditions (20.4 °C ± 1.7 °C and 60% ± 5% relative humidity).

On each test day, participants consumed 500 ml of water 2 h before testing to ensure euhydration. Upon arrival, participants voided their bladders and had their body weight measured. After instrumentation, participants ingested capsules containing either placebo or 6 mg/kg of caffeine (Sigma-Aldrich, Sydney, United States) with 50 mL of water (18, 24). One hour later, they entered a normobaric hypoxic tent (At-Home Cubicle Altitude Tent Standard, Hypoxico, United States) to prepare for the IST (see section “Intermittent-sprint test” below). A 60-min rest period followed capsule ingestion to allow sufficient time for blood caffeine levels to increase (25). All exercise tests were carried out on an electronically braked cycling ergometer (Lode Excaliber Sport, Groningen, Netherlands). Given the menstrual cycle does not meaningfully affect sprint performance and caffeine pharmacokinetics (26, 27), female participants were tested during the follicular phase as a conservative standardization strategy to minimize potential hormonal variability.

### Assessment of peak oxygen uptake and familiarization session

V̇O_2peak_ was assessed using an incremental cycling test previously implemented by our group (24). Expired gases were analyzed using a breath-by-breath gas analyzer (Metalyzer^®^3B; Cortex, Leipzig, Germany). The V̇O_2peak_ was defined as the highest 30-s mean value attained before volitional exhaustion. Power output and V̇O_2_ data from this test were used to set IST exercise intensity (28). After a 20-min recovery period, participants completed a 20-min familiarization IST, consisting of ten 2-min blocks (8-s sprint, 100-s active recovery at 35% V̇O_2peak_, and 12-s passive rest).

### Altitude simulation

A normobaric hypoxic tent (183 cm × 245 cm × 245 cm) was used to simulate ∼2,000 m altitude (inspired O_2_ fraction: 16.5% ± 0.2%) via a hypoxic gas generator (HYP123 Altitude Generator, Hypoxico, United States) (18). We chose moderate hypoxic conditions to reflect terrestrial altitude exposures (i.e., 1,800–2,200 m), such as Mexico City (∼2,240 m), which will host matches during the 23^rd^ FIFA World Cup in 2026, and which players may encounter during training and competition (2, 29, 30).

### Supplementation protocol

Caffeine doses were calculated based on body mass. Placebo capsules were filled with sugar alcohol (mannitol), which does not affect performance (31). Caffeine capsules contained pure caffeine powder. Participants ingested 3–5 identical gelatin capsules per trial, with the number standardized per individual. Blinding was maintained by an independent researcher who assigned alphanumeric codes, keeping both participants and investigators unaware of capsule contents.

### Intermittent-sprint test

Participants completed a standardized warm-up of 3 min cycling at 50% V̇O_2peak_, followed by two 1-min blocks alternating 30 s at 70% V̇O_2peak_ with 30 s rest. After 2 min seated rest, the IST began, consisting of two 40-min halves separated by 15 min of recovery (32). Each half included twenty 2 min-blocks, each comprising a 8-s “all-out” sprint, 100 s active recovery at 35% V̇O_2peak_, and 12 s passive recovery. After sprints 8 and 16, participants performed 5 × 4-s “all-out” sprints with 16 s of active recovery (35% V̇O_2peak_) to mimic repeated sprint bouts typical in team-sports (32, 33). All sprints were performed in “Wingate mode” on a cycle ergometer (LEM module Wingate Test, Lode, Groningen, Netherlands) with fixed resistance at 0.7 Nm/kg. The cycle ergometer had toe-clips to prevent foot slippage (18, 20). Participants were allowed 200 ml of room-temperature water at halftime (34).

### Measurements

#### Mechanical output

Mechanical outputs during the IST were recorded using the Lode Ergometry Manager Software (Version 10, Netherlands). Peak power output (PPO) was defined as the highest power output achieved during each sprint, mean power output (MPO) as the average power output over each 8-s sprint. Total work done was calculated as the sum of forty 8-s sprints and twenty 4-s sprints, excluding the active recovery periods.

#### Physiological responses

Respiratory gas variables, including O_2_ uptake, carbon dioxide (CO_2_) production, respiratory exchange ratio, minute ventilation, tidal volume, respiratory frequency, end-tidal CO_2_ and O_2_ pressures, as well as ventilatory equivalent for O_2_ and CO_2_ were measured breath-by-breath during the IST using a metabolic cart (Metalyzer 3B; Cortex, Leipzig, Germany). The system was calibrated immediately before each trial with a precision gas mixture (15% O_2_, 5% CO_2_) and a 3-L syringe for the flow meter. To prevent CO_2_ accumulation and control relative humidity, a two-way T-Shape non-rebreathing valve (Series 2700, Hans Rudolph, Kansas City, United States) was connected directly to the turbine flow sensor of the face mask to evacuate exhaled air from the tent.

Heart rate was recorded using a heart rate monitor (Vantage NV, POLAR, Kempele, Finland). Peripheral O_2_ saturation (SpO_2_) was continuously monitored with a fingertip pulse oximeter (Nellcor PM10N, Medtronic, MN, United States). Blood lactate was analyzed from samples collected before and within one hour after capsule ingestion, as well as immediately after each IST half, using a lactate analyzer (Biosen, Cline, Germany). Ratings of overall perceived exertion (Borg 6–20 scale), breathing difficulty, and lower-limb heaviness (Borg CR10 scale) were assessed after every fifth sprint (∼every 10 min) (35, 36).

### Statistical analyses

Data are presented as means ± SD. Two-way repeated-measures ANOVA [time (first vs. second half and sprint number: 1–40) × condition (caffeine and placebo)] was used to analyze total PPO, MPO, and total work done. Perceptual responses and blood lactate concentration were analyzed using two-way repeated-measures ANOVA [time (sprint number: 5, 10, …, 40) or testing points (pre, post, first half vs. second half) × condition (caffeine vs. placebo)]. Cardiorespiratory variables and SpO_2_ were also analyzed using two-way repeated-measures ANOVA [stage (sprint block: 1–5, 6–10, …, 36–40) × condition (caffeine vs. placebo)]. Sphericity was assessed using Mauchly’s test, and where the assumption of sphericity was violated, the Greenhouse-Geisser correction was applied. Significant main or interaction effects were followed by paired-samples *t*-tests with Bonferroni adjustments. Percent changes in total work done between trials and halves were analyzed using paired *t*-tests corrected for Bonferroni multiple comparisons. Effect sizes were reported as partial eta-squared (ηp^2^) for main effects and Hedges’ *g* for pairwise comparisons (37). Analyses were performed using SPSS v28 (IBM, United states), with statistical significance set at *p* ≤ 0.05.

## Results

### Prolonged intermittent-sprint performance

**FIGURE 1:**
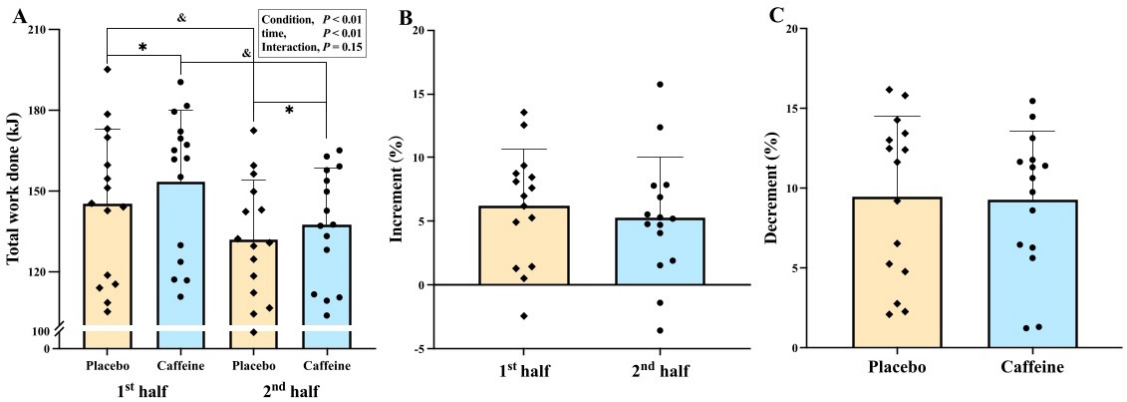
Total work done **(A)**, percent increase **(B)** and percent decrement **(C)** during the intermittent-sprint test. *p < 0.05 vs. Placebo, ^&^*p* < 0.05 vs. First half.

Total work showed significant main effects of condition (*F* = 11.205, *p* < 0.01, ηp^2^ = 0.445) and time (*F* = 14.45, *p* < 0.01, ηp^2^ = 0.508) (Figure 1). *Post hoc* analysis revealed a decline in total work done from the first to second half for both caffeine (*p* < 0.05, *g* = 0.24) and placebo (*p* < 0.05, *g* = 0.25), with no difference in percent decrement between conditions (9.3% vs. 9.5%, *p* > 0.05, *g* = 0.20). Caffeine increased total work done compared with placebo in both the first (*p* < 0.01, *g* = 0.71) and second half (*p* = 0.017, *g* = 0.64), with no significant difference in percent increase between halves (6.2% vs. 5.3%, *p* > 0.05, *g* = 0.16).

**FIGURE 2:**
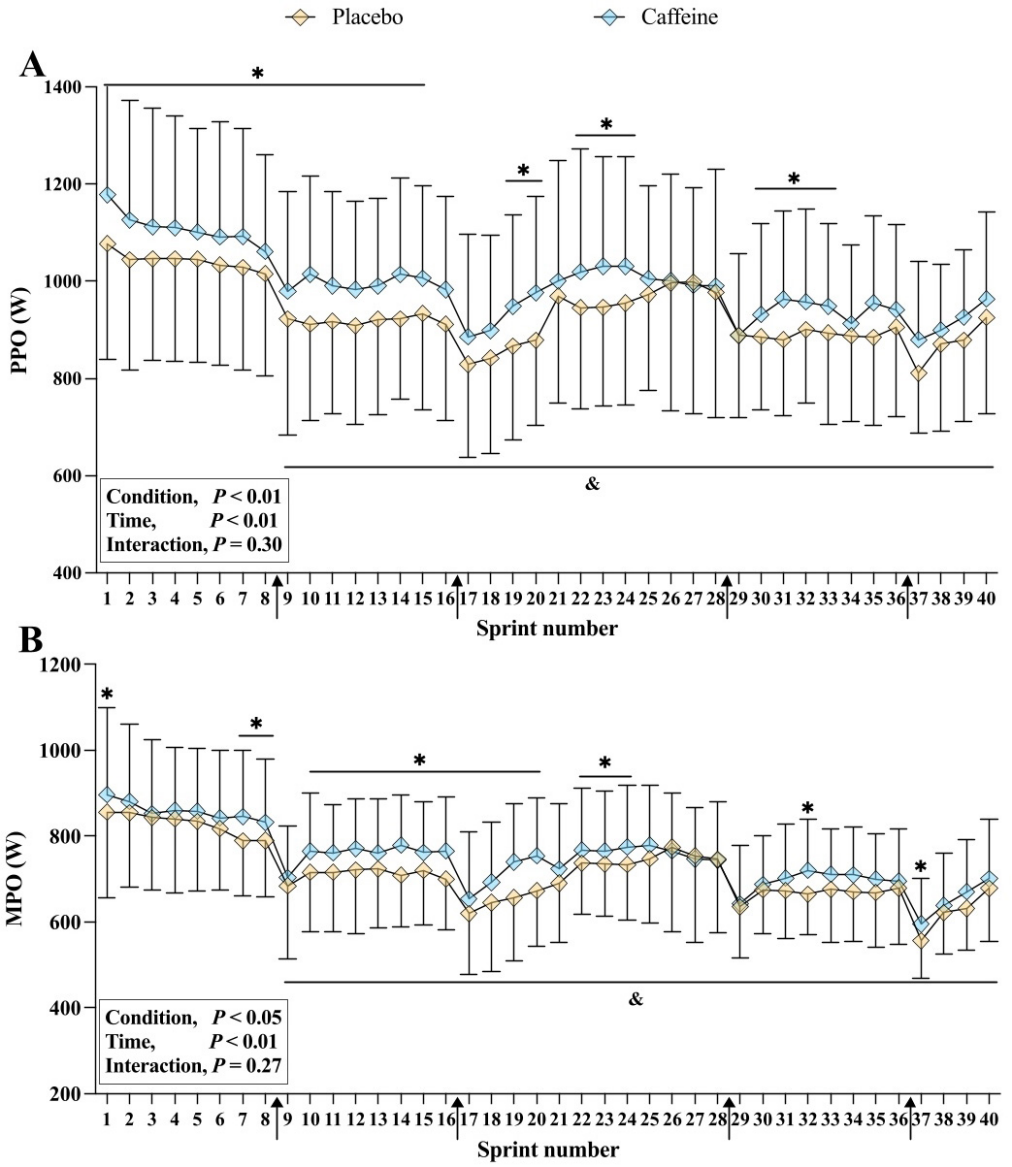
Peak power output (PPO) **(A)** and mean power output (MPO) **(B)** during the intermittent-sprint test. *p < 0.05 vs. Placebo, ^&^*p* < 0.05 vs. Sprint number 1, ↑Repeated-sprint bout.

A significant main effect of condition (*F* = 7.830, *p* < 0.05, ηp^2^ = 0.359) and time (*F* = 12.607, *p* < 0.01, ηp^2^ = 0.474) was found for PPO (Figure 2A). Specifically, PPO was higher with caffeine than placebo during the sprint number 1–15, 19–20, 22–24, and 30–32 (all *p* < 0.05, *g* = 0.29–0.35). For MPO, significant main effects of condition (*F* = 9.411, *p* < 0.01, ηp^2^ = 0.402) and time (*F* = 22.203, *p* < 0.01, ηp^2^ = 0.613) were also noted (Figure 2B). Specifically, MPO were higher with caffeine than placebo during the sprint number 1, 6–20, 23–25, 32 and 37 (all *p* < 0.05, *g* = 0.24–0.36). Both PPO and MPO decreased progressively across repeated sprints (all *p* < 0.05, Figures 2A, B).

### Blood lactate concentration

**FIGURE 3:**
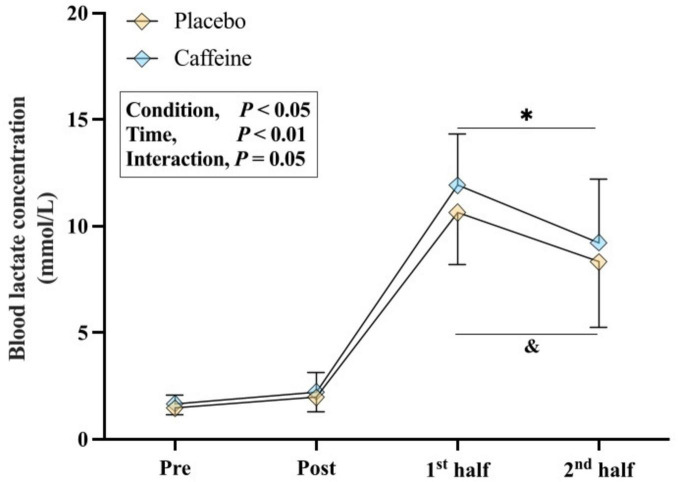
Blood lactate concentration measured pre- and within one hour post-capsule intake at rest, and immediately after the first and second halves. *p < 0.05 vs. Placebo, ^&^*p* < 0.05 vs. Pre.

Significant main effects were observed for condition (*F* = 6.259, *p* = 0.022, ηp^2^ = 0.248) and time (*F* = 15.146, *p* < 0.01, ηp^2^ = 0.444) (Figure 3). Blood lactate concentrations after both the first and second halves were higher with caffeine than placebo (all *p* < 0.05, *g* = 0.30–0.35).

### Perceptual and physiological responses

**FIGURE 4:**
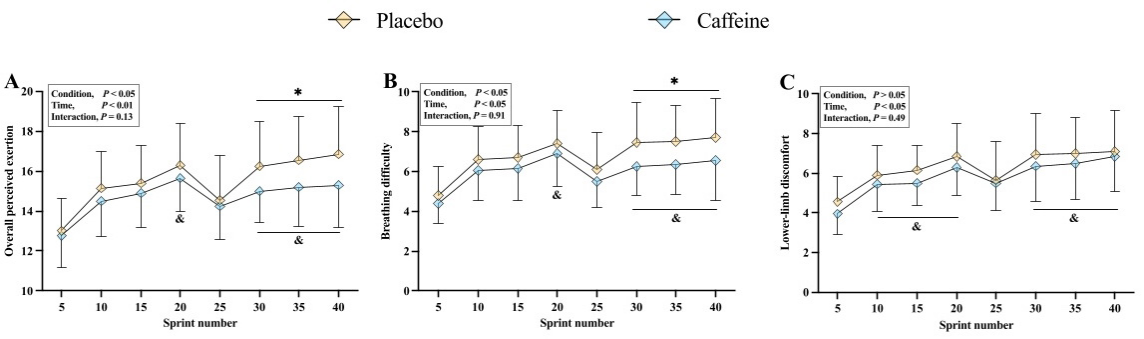
Ratings of perceived exertion for overall rating of perceived exertion **(A)**, breathing difficulty **(B)** and lower-limb heaviness **(C)** during the intermittent-sprint test. *p < 0.05 vs. Placebo, ^&^*p* < 0.05 vs. Sprint number 5.

Significant main effects of condition were observed for overall perceived exertion (*F* = 6.361, *p* = 0.021, ηp^2^ = 0.251) and breathing difficulty (*F* = 10.780, *p* < 0.01, ηp^2^ = 0.362), but not for lower-limb discomfort (*F* = 2.111, *p* > 0.05, ηp^2^ = 0.100). All perceptual ratings increased progressively over time (all *p* < 0.01) (Figures 4A–C). Compared with placebo, caffeine reduced overall perceived exertion and breathing difficulty during the sprint number 30, 35, and 40 (all *p* < 0.05, *g* = 0.25–0.46).

There were a significant main effect of condition on minute ventilation, tidal volume, heart rate, ventilatory equivalents for O_2_ and CO_2_, end-tidal O_2_ and CO_2_ partial pressures during the IST (all *p* < 0.05). Compared with placebo, caffeine increased minute ventilation (sprint block 6–20), tidal volume (36–40), heart rate (11–20 and 26–40), ventilatory equivalent for O_2_ (1–10 and 16–40), ventilatory equivalent for CO_2_ (16–40), and end-tidal O_2_ partial pressure (11–40) (all *p* < 0.05), while reducing end-tidal CO_2_ partial pressure (11–35) (all *p* < 0.05). All variables also demonstrated a significant main effect of time (all *p* < 0.001), with most increasing progressively across sprints except, for SpO_2_ and end-tidal CO_2_ partial pressure, which decreased (all *p* < 0.05) (Table 1).

**TABLE 1:** Cardiorespiratory variables during intermittent-sprint test with and without caffeine consumption.

Variables	Condition	Sprint blocks								ANOVA *p*-values		
		1–5	6–10	11–15	16–20	21–25	26–30	31–35	36–40	Condition	Stage	Interaction
O_2_ uptake (L/min) CO_2_ production (L/min)	Placebo	2.07 ± 0.36	2.25 ± 0.50^&^	2.15 ± 0.38	2.17 ± 0.49	1.99 ± 0.37	2.33 ± 0.35^&^	2.23 ± 0.38^&^	2.29 ± 0.49^&^	0.566 0.110	<0.01 <0.01	0.887 0.220
	Caffeine	2.02 ± 0.36	2.29 ± 0.43^&^	2.20 ± 0.42^&^	2.28 ± 0.44^&^	2.06 ± 0.55	2.36 ± 0.36^&^	2.22 ± 0.40^&^	2.37 ± 0.46^&^			
	Placebo	2.38 ± 0.39	2.62 ± 0.52^&^	2.68 ± 0.37^&^	2.19 ± 0.46	1.90 ± 0.33	2.67 ± 0.37^&^	2.57 ± 0.38	2.52 ± 0.42			
	Caffeine	2.37 ± 0.43	2.62 ± 0.45^&^	2.67 ± 0.41^&^	2.71 ± 0.44^&^	2.01 ± 0.28	2.76 ± 0.33^&^	2.74 ± 0.33^&^	2.84 ± 0.39^&^			
Minute ventilation (L/min)	Placebo	82 ± 15	98 ± 20^&^	93 ± 18^&^	92 ± 20^&^	74 ± 13	93 ± 19^&^	88 ± 20^&^	90 ± 18^&^	0.014	<0.01	0.938
	Caffeine	88 ± 17	105 ± 17^*&^	104 ± 18^*&^	104 ± 17^*&^	84 ± 19	100 ± 21^&^	95 ± 18^&^	97 ± 20^*&^			
Tidal volume (L)	Placebo	1.40 ± 0.24	1.56 ± 0.34^&^	1.55 ± 0.33^&^	1.53 ± 0.33^&^	1.34 ± 0.28	1.52 ± 0.34^&^	1.54 ± 0.39^&^	1.57 ± 0.34^&^	<0.01	<0.01	0.110
	Caffeine	1.44 ± 0.31	1.71 ± 0.37^*&^	1.70 ± 0.35^*&^	1.71 ± 0.41^*&^	1.45 ± 0.31	1.63 ± 0.26^&^	1.49 ± 0.28^&^	1.68 ± 0.28^*&^			
Breathing frequency (breaths/min)	Placebo	57 ± 11	63 ± 12^&^	61 ± 12	63 ± 13^&^	55 ± 12	62 ± 13	64 ± 15^&^	65 ± 14^&^	0.167	<0.01	0.050
	Caffeine	57 ± 24	61 ± 14	65 ± 14^&^	64 ± 14^&^	61 ± 17	64 ± 14^&^	66 ± 15^&^	68 ± 15^&^			
Respiratory exchange ratio	Placebo	1.17 ± 0.08	1.12 ± 0.07	1.06 ± 0.05^&^	1.04 ± 0.05^&^	0.95 ± 0.05^&^	1.02 ± 0.07^&^	0.98 ± 0.05^&^	0.98 ± 0.06^&^	0.212	<0.01	0.828
	Caffeine	1.19 ± 0.08	1.15 ± 0.07	1.09 ± 0.07^&^	1.06 ± 0.04^&^	0.97 ± 0.06^&^	1.05 ± 0.06^&^	1.02 ± 0.06^&^	1.00 ± 0.05^&^			
Ventilatory equivalent for O_2_	Placebo	35.8 ± 3.1	38.9 ± 4.1^&^	39.5 ± 3.8^&^	36.9 ± 3.1^&^	32.7 ± 4.1	35.6 ± 4.6	38.4 ± 4.7^&^	34.8 ± 4.4	<0.01	<0.01	0.464
	Caffeine	36.1 ± 5.4*	40.5 ± 4.9^*&^	40.2 ± 7.9^&^	39.5 ± 4.2^*&^	36.4 ± 6.3*	38.6 ± 5.4^*&^	40.7 ± 5.4^*&^	37.0 ± 4.3*			
Ventilatory equivalent for CO_2_	Placebo	30.9 ± 2.7	34.3 ± 3.3^&^	36.2 ± 3.8^&^	35.7 ± 3.9^&^	34.8 ± 4.7^&^	35.1 ± 4.5^&^	36.1 ± 4.6^&^	35.2 ± 4.4^&^	<0.01	<0.01	0.152
	Caffeine	32.2 ± 4.3	36.2 ± 4.4^*&^	36.1 ± 7.1^&^	37.4 ± 4.9^*&^	37.5 ± 6.2^*&^	37.1 ± 5.8^*&^	38.2 ± 5.6^*&^	37.3 ± 5.1^*&^			
End-tidal O_2_ partial pressure (mmHg)	Placebo	86.2 ± 2.3	92.9 ± 1.7^&^	92.1 ± 1.1^&^	92.5 ± 1.3^&^	88.1 ± 2.7	91.1 ± 1.7	91.0 ± 1.4	90.1 ± 2.7	<0.01	<0.01	0.079
	Caffeine	87.5 ± 1.9	94.1 ± 1.3^&^	94.8 ± 0.8^*&^	94.1 ± 1.1^*&^	91.0 ± 2.3*	93.0 ± 1.1^*&^	93.4 ± 0.8^*&^	92.6 ± 1.0^*&^			
End-tidal CO_2_ partial pressure (mmHg)	Placebo	35.8 ± 2.0	31.3 ± 1.6^&^	31.5 ± 0.8^&^	31.0 ± 1.0^&^	32.7 ± 2.1	32.5 ± 1.3	31.8 ± 0.9^&^	31.7 ± 2.0^&^	0.024	<0.01	0.296
	Caffeine	34.3 ± 1.8	31.1 ± 1.2^&^	29.1 ± 0.9^*&^	29.9 ± 1.1^*&^	30.7 ± 1.9^*&^	31.2 ± 1.0^*&^	30.2 ± 0.7^*&^	31.0 ± 1.0^&^			
Heart rate (beats/min)	Placebo	158 ± 11	172 ± 7^&^	173 ± 8^&^	174 ± 8^&^	167 ± 7	176 ± 8^&^	176 ± 8^&^	179 ± 7^&^	0.044	<0.01	0.053
	Caffeine	161 ± 15	172 ± 13^&^	179 ± 13^*&^	181 ± 13^*&^	169 ± 13	176 ± 14^*&^	182 ± 12^*&^	183 ± 13^*&^			
SpO_2_ (%)	Placebo	96 ± 2	94 ± 2^&^	91 ± 3^&^	90 ± 4^&^	92 ± 2^&^	89 ± 3^&^	89 ± 3^&^	88 ± 4^&^	0.104	<0.01	0.588
	Caffeine	96 ± 3	94 ± 4^&^	92 ± 4^&^	91 ± 3^&^	93 ± 2	90 ± 3^&^	90 ± 4^&^	89 ± 3^&^			

Data are means ± SD, SpO_2_: arterial O_2_ saturation, *p < 0.05 vs. Placebo, ^&^*p* < 0.05 vs. Sprint block 1–5.

## Discussion

### Summary of main findings

This study is the first to examine the effects of caffeine intake before exercise on prolonged intermittent-sprint performance in team-sport athletes under moderate normobaric hypoxia using a validated IST protocol that replicates match demands. Consistent with our hypothesis, caffeine significantly enhanced total work done, PPO and MPO. Additionally, caffeine increased heart rate, minute ventilation, and blood lactate concentrations, while reducing ratings of overall perceived exertion and breathing difficulty during second half compared to placebo. Other cardio-respiratory parameters remained unchanged. These findings support caffeine as an effective ergogenic aid for enhancing prolonged intermittent exercise performance in moderate hypoxic conditions relevant to team-sports.

### Prolonged intermittent-sprint performance

In the caffeine trial, total work done during the IST (2 × 40 min) was 6.2% higher in the first half (*g* = 0.71) and 5.3% higher in the second half (*g* = 0.64) compared to placebo under normobaric hypoxia (Figure 1). Hypoxia-related impairments in multiple sprints are preliminary linked to changes in energy metabolism and neuromuscular function (1, 3). Our results align with previous research using a similar protocol (2 × 36 min) and caffeine dose (6 mg/kg) under normoxia, which reported comparable improvements (7.6%–8.5%; *g* = 0.50–0.46) (20). This suggests caffeine’s ergogenic benefits extend to prolonged intermittent-sprint performance in both normoxic and hypoxic environments. Several mechanisms may explain these performance enhancements. Caffeine, as an adenosine receptor antagonist, stimulates the central nervous system, thereby enhancing motor unit recruitment and/or firing frequency (38, 39). This is partially supported by higher PPO and MPO values at iso-sprint numbers during the IST in the caffeine condition (Figure 2). In addition, caffeine may directly influence skeletal muscle by attenuating interstitial potassium accumulation through increased Na^+^-K^+^ pump activity (40) and by promoting calcium release from the sarcoplasmic reticulum or enhancing its activation within muscle fibers (41), thereby facilitating excitation-contraction coupling and increasing muscle contractility (42).

Previous studies suggest caffeine may enhance anaerobic energy supply, indicated by elevated blood lactate concentrations (43), thereby improving exercise capacity under hypoxic conditions (18, 24). In this study, despite no significant differences in O_2_ uptake between conditions (Table 1), higher blood lactate concentrations with caffeine (Figure 3) suggest increased anaerobic glycolysis contributed to enhanced prolonged intermittent-sprint performance in hypoxia. It should also be acknowledged that the higher blood lactate concentrations observed with caffeine may partly reflect enhanced lactate efflux from working muscle secondary to caffeine-induced hyperventilation and respiratory alkalosis, rather than greater intramuscular lactate production *per se*. Consequently, when respiratory alkalosis is present, blood lactate responses should be interpreted cautiously as an index of anaerobic energy contribution. Additionally, greater reliance on anaerobic metabolism can cause inorganic phosphate accumulation and reduced muscle pH, thereby promoting peripheral fatigue (44, 45). This may partly explain why caffeine significantly enhanced power output at iso-sprint numbers, with effects most pronounced in the first half of the IST (Figure 2), consistent with previous research (46). In addition, the glycogen-sparing theory, first proposed by Costill (47), suggests that caffeine improves endurance performance by increasing free fatty acids and adrenaline during submaximal exhaustive cycling (47, 48). However, future studies should incorporate blood triglyceride assessments to determine whether this assumption extends to prolonged intermittent-sprint performance.

Our previous findings have demonstrated that the ventilatory response is crucial for maintaining exercise performance under hypoxia (24, 49, 50). Increased minute ventilation enhances oxygen intake, raises arterial O_2_ content, and improves O_2_ delivery to skeletal muscles (51, 52). Greater O_2_ availability may also facilitate phosphocreatine (PCr) resynthesis, thereby contributing to improved multiple sprint performance in hypoxia (53). Our results show that caffeine increased minute ventilation, and although SpO_2_ did not significantly increase, an upward trend was observed (Table 1). Future studies utilizing near-infrared spectroscopy to assess muscle and/or cerebral oxygenation during IST is needed to further test this hypothesis. Caffeine is well known to modulate perceived exertion, enhancing exercise performance by either increasing mechanical output at the same effort or reducing effort at a given intensity (14, 54). Our findings on perceptual responses (overall perceived exertion, breathing difficulty, and lower-limb heaviness) support this effect of caffeine (Figure 4).

Another notable finding was the large inter-individual variability in the percentage change in total work done (first half: −2% to −14%; second half: −4% to −16%; Figure 1B). Since participants were naïve-to-mild caffeine consumers, these differences may be linked to genetic variations and/or potential sex differences (55, 56). The CYP1A2 genotype (AA vs. AC/CC) plays a key role in modulating the ergogenic effects of caffeine (57). In addition, Paton et al. (58) found caffeine enhanced sprint power to a greater extent in male than female during 30-km cycle time trials. However, more research is required to clarify how genetics and sex affect the ergogenic effect of caffeine in team-sport athletes.

### Limitations and additional considerations

Several limitations should be acknowledged when interpreting these findings. The IST was performed on a cycle ergometer, which provides a controlled and reproducible model of repeated high-intensity efforts but lacks the biomechanics, muscle recruitment, and metabolic demands of running-based team-sports. Moreover, the study was conducted under moderate normobaric hypoxia (∼2,000 m equivalent) in a hypoxic tent, which does not fully replicate terrestrial altitude or account for reduced air resistance that can actually increase single sprint performance (2). Responses at higher altitudes (2,000–3,600 m), where oxygen delivery is more severely compromised, or at low altitude (<2,000 m), where hypoxic stress is limited, may differ from those observed here. Therefore, caution is warranted when generalizing these results to real-world, field-based settings.

Mechanistic interpretation is limited by the absence of muscle and/or cerebral oxygenation measurements using near-infrared spectroscopy, a key determinant of sprint capacity under hypoxia (59), and the lack of neuromuscular assessments (60) that could have provided additional insight into fatigue development. Previous work suggests that caffeine may improve performance primarily through reduced perception of effort rather than neuromuscular alterations (61), which aligns with the lower exertion ratings observed here. Additionally, despite instructions to sprint maximally, the prolonged protocol may have encouraged pacing strategies, potentially dampening physiological differences and masking the full ergogenic effect of caffeine. Finally, as the participants were university-level athletes, the generalizability of the findings may be limited (62). Future research should include better-trained athletes to more thoroughly investigate the ergogenic effects of caffeine.

### Practical applications

Our findings indicate that caffeine intake before exercise enhances prolonged intermittent-sprint performance in a simulated team-sport setting during an 80-min protocol under hypoxic conditions. Moreover, our previous research demonstrated that moderate caffeine doses (6 mg/kg) do not increase the risk of side effects such as headache, gastrointestinal discomfort, or insomnia (18). Therefore, we recommend that athletes in team-sports such as football, rugby, and hockey consider moderate caffeine supplementation when competing at moderate altitudes, as it may confer a competitive advantage.

## Conclusion

Moderate caffeine intake before exercise enhances performance in a prolonged intermittent-sprint test simulating the physiological demands of team-sport athletes in normobaric hypoxia. These findings offer practical guidance for caffeine supplementation strategies when preparing to compete at moderate altitude.

## Data Availability

The original contributions presented in this study are included in this article/supplementary material, further inquiries can be directed to the corresponding author.
